# Causes and management strategies for elevated intraocular pressure after implantable collamer lens implantation

**DOI:** 10.3389/fmed.2024.1351272

**Published:** 2024-02-07

**Authors:** Di Gong, Simin Deng, Kuanrong Dang, Zonghui Yan, Jiantao Wang

**Affiliations:** ^1^Shenzhen Eye Hospital, Jinan University, Shenzhen Eye Institute, Shenzhen, Guangdong, China; ^2^The 2nd Clinical Medical College (Shenzhen People's Hospital) of Jinan University, Shenzhen, China

**Keywords:** intraocular pressure, implantable collamer lens, complications, management strategies, ophthalmic surgery

## Abstract

With the widespread application of Implantable Collamer Lens (ICL) implantation surgery in the field of myopia correction, a comprehensive understanding of its potential complications, especially those related to intraocular pressure (IOP), becomes crucial. This article systematically reviews various complications that may lead to IOP elevation after ICL surgery. Firstly, common complications after ICL surgery, including residual viscoelastic, steroid response, and excessive vault of the ICL, are detailed, emphasizing their potential impact on intraocular pressure. Regarding residual viscoelastic, we delve into its direct relationship with postoperative elevated IOP and possible preventive measures. For steroid response, we stress the importance of timely adjustment of steroid therapy and monitoring intraocular pressure. Additionally, excessive vault of the ICL is considered a significant potential issue, and we elaborate on its mechanism and possible management methods. In further discussion, we focus on relatively rare complications such as Toxic Anterior Segment Syndrome (TASS), Urrets-Zavalia Syndrome (UZS), Pigment Dispersion Syndrome (PDS), and malignant glaucoma. For these relatively rare complications, this review thoroughly explores their potential mechanisms, emphasizes the importance of prevention, and provides guidance for early diagnosis and treatment. This is a comprehensible review that aims to offer eye care professionals a comprehensive understanding and effective management guidance for complications of elevated IOP after ICL surgery, ultimately providing optimal care for patients’ visual health.

## 1 Introduction

Implantable Collamer Lens (ICL), a type of intraocular lens used for correcting refractive errors, can be implanted between the iris and crystalline lens. It is widely employed for correcting various degrees of refractive errors, including myopia, hyperopia, and astigmatism. ICL as also used to treat irregular astigmatism in etcatic corneal disorders such as keratoconus (1). ICL implantation surgery with a crystalline lens offers excellent visual correction, relatively low risk, reversibility, and a broad range of applicability. It is suitable not only for patients who do not meet the conditions for corneal laser surgery but also provides additional correction options for those with a desire for spectacle independence (2).

Before the clinical application of the V4c lens, patients undergoing ICL surgery (such as V4a, V4b) required YAG laser peripheral iridotomy before implantation to facilitate postoperative aqueous humor circulation. The new generation ICL V4c, however, incorporates three very small holes in the lens design. The central hole of the ICL V4c not only allows smoother aqueous humor flow from the posterior to the anterior chamber, effectively preventing postoperative elevated intraocular pressure (3). Simultaneously, the presence of the central hole, by providing more natural aqueous humor circulation around the lens, may help reduce the occurrence of postoperative cataracts (4–6). Therefore, the widespread use of V4c effectively reduces the occurrence of complications after ICL implantation (7).

Despite being considered a safe and effective method for refractive correction, ICL surgery is associated with a variety of postoperative complications. Common complications include abnormal vault of the lens, malpositioning of the lens, loss and decompensation of corneal endothelial cells, elevated intraocular pressure (IOP), cataract formation, and night vision symptoms (8). Among these, elevated IOP accounts for approximately 10.8% of post-ICL complications, presenting with symptoms such as eye bulging, eye pain, and even systemic symptoms like headache and nausea. Common causes of post-ICL elevated IOP include steroid response, residual viscoelastic, pupil block, iris pigment deposition, and narrow anterior chamber angle (ACA) (9). Early detection of signs of elevated IOP and appropriate interventions can minimize the damage associated with IOP elevation. Therefore, understanding the causes and management strategies of elevated IOP after ICL surgery is of paramount importance. This paper is a comprehensible review of the common causes of elevated IOP after ICL implantation and their management strategies, with a view to providing guidance to surgeons performing ICL surgery.

## 2 Residual ophthalmic viscosurgical device

The initial surge in IOP following ICL implantation occurs on the first day postoperatively, primarily due to the mechanical obstruction of the trabecular meshwork caused by residual ophthalmic viscosurgical devices (OVD) (9). OVDs are commonly utilized in intraocular surgeries to maintain anterior chamber stability and protect corneal endothelial cells during the surgical process (10). Early postoperative elevated IOP due to residual viscosurgical substance in ICL surgery often presents with unbearable eye pain and corneal epithelial edema. The measured IOP peaks are commonly at 30 mmHg or higher, posing a potential risk of retinal artery occlusion and anterior ischemic optic neuropathy. To prevent postoperative IOP elevation related to viscosurgical residue, a crucial step is the thorough removal of OVD during surgery.

Currently, commonly used viscosurgical substances in ophthalmology include sodium hyaluronate (HA) and hydroxypropyl methylcellulose (HPMC). Studies suggest that HA, characterized by high cohesiveness and dispersion, is challenging to completely flush from the anterior chamber, making it more likely to cause postoperative elevated IOP. On the other hand, HPMC, mainly possessing viscosity, is relatively easier to remove from the anterior chamber (9). However, a study by Ganesh S et al. compared the effects of using 2% HPMC and 1% HA as viscosurgical agents during ICL surgery on postoperative IOP and surgical time. The results indicated that, compared to 2% HPMC, the HA group had a shorter total surgical time and a lower incidence of acute elevated IOP (11). Due to the current lack of comparative research between the two types of OVDs in ICL, further evidence from evidence-based medicine is needed to guide the choice of viscosurgical substances.

**Figure 1:**
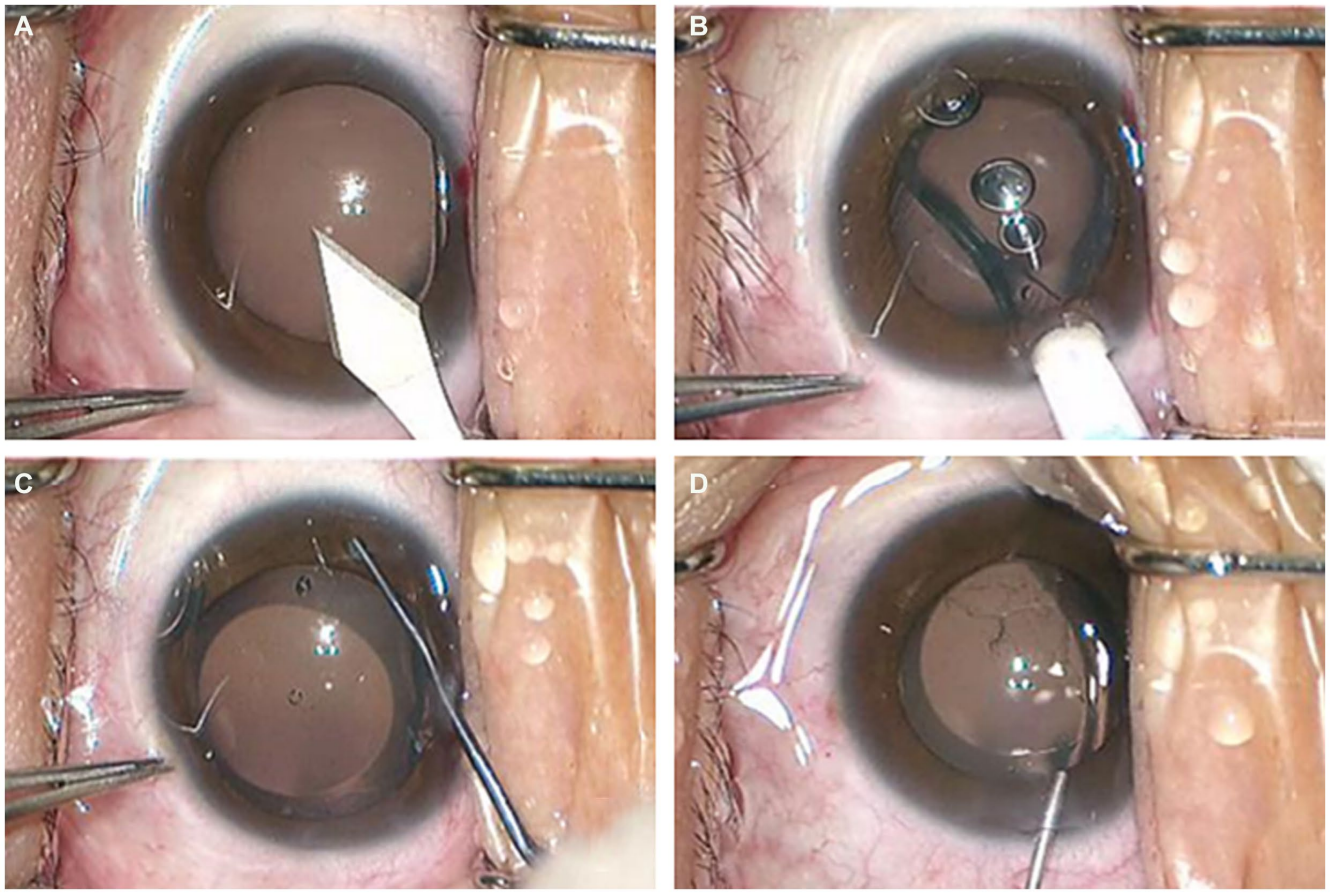
The streamlined steps for the operation of the min-OVD technique. **(A)** incision in the temporal transparent cornea; **(B)** implantation of ICL; **(C)** injection of the OVD into the anterior chamber and adjust the lens to proper position; **(D)** thoroughly flushing the OVD.

The application of OVD in traditional ICL implantation is known as the two-step OVD technique, involving two injections of OVD into the anterior chamber during the surgery. The first injection occurs after completing the corneal incision, where the OVD is injected to maintain anterior chamber stability. The second injection is performed after implanting the ICL lens, aiming to protect corneal endothelial cells from mechanical damage while adjusting the ICL to the posterior chamber. Subsequently, balanced salt solution (BSS) is used to flush the OVD after placing the ICL in the appropriate position (12). Due to limited operating space, OVD residue that has entered the posterior chamber may be challenging to completely wash out, leading to trabecular meshwork blockage and, in severe cases, causing pupil-blocking glaucoma. The streamlined steps for the operation of the min-OVD technique are shown in Figure 1. In recent years, experienced surgeons have introduced a one-step viscosurgical device technique, also known as the minimum ophthalmic viscosurgical device (min-OVD) technique, to address the issue of posterior chamber OVD residue. The min-OVD technique skips the first OVD injection, immediately implanting the ICL after completing the corneal incision. The viscosurgical substance is then injected between the ICL and corneal endothelium, followed by washing the OVD after adjusting the ICL to its proper position. This technique prevents OVD from entering the posterior chamber, significantly reduces the difficulty of thoroughly flushing the OVD, thereby decreasing the occurrence of postoperative elevated intraocular pressure (IOP) and improving surgical quality (13, 14).

Building on the min-OVD technique, some surgeons have proposed the non-ophthalmic viscosurgical device (non-OVD) technique. This approach completely avoids the use of OVD and BSS while maintaining anterior chamber stability during surgery (15–17). Zhang Z et al. compared the safety of min-OVD and non-OVD techniques for ICL implantation by evaluating visual outcomes, corneal endothelial cell density (ECD), and corneal densitometry at 1, 2, 3, and 24 h postoperatively. The results showed no statistically significant differences in visual outcomes between the two groups, while the non-OVD group had significantly shorter surgical times, and IOP at 1 and 2 h postoperatively was significantly lower than that in the min-OVD group (17). The non-OVD technique may be a safer method for ICL implantation as it completely eliminates ocular viscosurgical device-related complications. However, this method also forfeits the positive effects of viscosurgical substances, potentially leading to issues such as anterior chamber disappearance, operational challenges, and loss of corneal endothelial cells. Therefore, surgeons may choose the OVD application method based on their experience.

Management Strategy: If patients experience symptoms such as eye swelling, headache, or nausea and vomiting within 24 h after ICL surgery, the possibility of postoperative OVD residue should be considered. After sufficient communication with the patient, removing the eye dressing and conducting slit-lamp observation and IOP measurement is recommended. If the IOP in the operated eye is only mildly elevated, short-term application of ocular hypotensive eye drops may be considered. If the intraocular pressure in the operated eye exceeds 30 mmHg and continues to rise, considering another anterior chamber washout is advisable to thoroughly remove the remaining viscosurgical substance, thus preventing further visual function damage caused by acute elevated IOP.

## 3 Steroid response

The second peak of elevated IOP after ICL surgery typically occurs between 1 to 4 weeks postoperatively. Steroid-induced ocular hypertension (SIOH) is the primary cause of IOP elevation after ICL surgery, accounting for approximately 64% of cases due to routine local application of corticosteroid eye drops. The concept of SIOH is generally defined as an IOP increase of >10 mmHg compared to the baseline after corticosteroid use, with clinical significance. In the general population, SIOH is estimated to occur in approximately 15–30% of cases (9, 18, 19). SIOH may lead to further damage to the optic nerve and visual function, resulting in steroid-induced glaucoma (SIG) (20).

The exact mechanisms of SIOH and SIG are not fully understood, but a reduction in trabecular meshwork outflow is considered a major contributor to elevated IOP (18). Cortisol, as the most crucial human glucocorticoid, plays a vital role in stress responses and the regulation of natural feedback mechanisms, suppressing inflammatory reactions. Therefore, glucocorticoids have broad pharmacological applications for treating various diseases. Through membrane diffusion and binding to intracellular receptors, glucocorticoids initiate a cascade of signaling events, ultimately affecting the expression of hundreds of genes. This implies a highly individualized response potential to glucocorticoid treatment, including adverse reactions in susceptible patients. The results of the first polymorphic whole-genome association study designed to identify genetic variations related to SIOH revealed two new genes, GPR158 and HCG22, associated with the disease, offering prospects for prediction and diagnosis (20). In addition to specific genetic mutations, various risk factors have been identified, primarily including a personal or family history of primary open-angle glaucoma (POAG), with the type, route of administration, dosage, and duration of treatment also playing crucial roles (19, 21). The Precision Medicine Initiative announced by President Obama in the 2015 State of the Union address outlined how it would extend to ophthalmic practice, presenting an opportunity for the effective application of precision medicine in SIOH/SIG (20).

Management Strategy: High-risk patients receiving corticosteroid therapy after ICL surgery should be closely monitored. If elevated eye pressure occurs between 1 to 4 weeks postoperatively, discontinuation or replacement with a lower-potency corticosteroid is recommended, accompanied by the topical application of glaucoma medications. In most SIOH patients, eye pressure typically returns to normal within 1 to 4 weeks after discontinuing steroids. However, approximately 1–5% of patients show no response to medication and require further glaucoma surgical intervention. The most commonly used procedure is trabeculectomy, but drainage device implantation or cyclodestructive surgery can also be considered (19).

## 4 Excessive ICL vault

**Figure 2:**
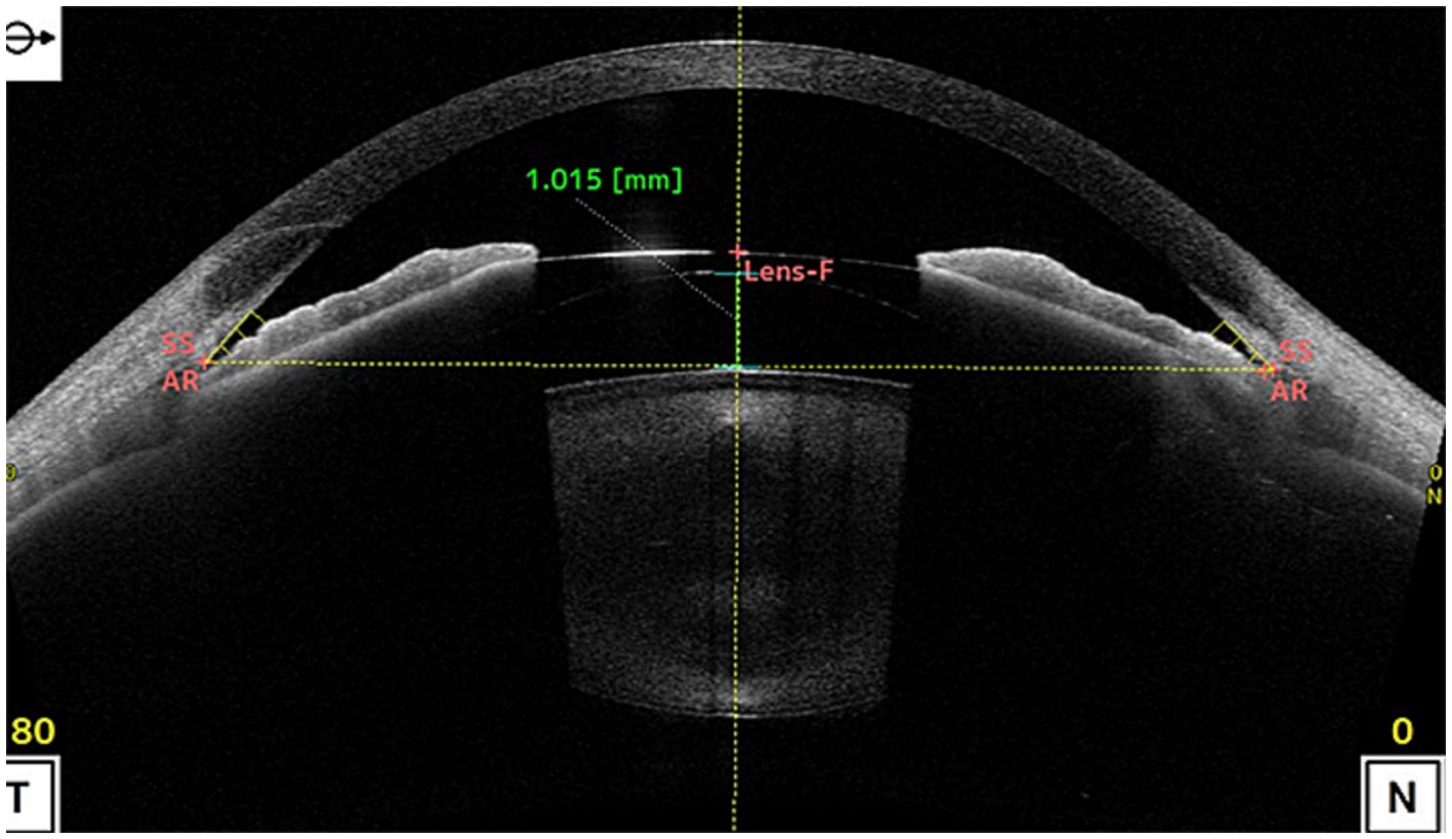
shows the vault was as high as 1.015 mm after ICL implantation, causing stenosis of the anterior chamber angle. (SS, scleral spur; AR, angle recess; Lens-F, lens anterior surface intersection. The horizontal yellow dotted line is the SS connection line, and the vertical yellow dotted line is the mid-penetration line of the SS connection line. The green dotted line is the indicator line of the crystal vault).

ICL vault refers to the space between the ICL and the natural lens or iris of the eye. The height of the vault is an important factor in determining the postoperative results of ICL implantation surgery (22). If the vault is too low, it can lead to contact between the ICL and the natural lens or iris, causing potential complications such as cataract formation or pigment dispersion syndrome. On the other hand, if the vault is too high, it can lead to increased IOP and potential complications such as glaucoma (23, 24). Figure 2 shown the excessive vault after ICL implantation, causing stenosis of the anterior chamber angle. Previous studies have investigated the effect of ICL vault height on postoperative results, they found that a vault height of 250–750 microns is generally considered ideal for optimal postoperative outcomes. A vault within this range minimizes the risk of complications such as cataract formation and pigment dispersion syndrome while also reducing the risk of increased IOP (2).

Although the exact mechanism of IOP caused by excessive ICL vault needs to be further explored, existing studies suggest that may be related to mechanical compression, inflammation and pigment dispersion (25, 26). First, the excessive ICL vault can cause mechanical compression of the surrounding structures, including the ciliary body and the iris. This compression can directly cause the ciliary body obstruct and the anterior chamber angle narrow or closure, potentially affecting the production and drainage of aqueous humor, leading to IOP. Furthermore, excessive vault can also lead to inflammation and pigment dispersion syndrome, where inflammatory and pigment granules from the iris are released into the anterior chamber. These cause the aqueous humor outflow blockage and resistance, leading to IOP. IOP due to excessive ICL vault after surgery can present with various clinical signs, including anterior chamber shallowing, corneal edema, iridocorneal touch, even if glaucomatous optic nerve changes (27). When such signs appear after operation, we should consider the possibility of IOP caused by excessive ICL vault.

Various factors can affect the position and stability of the ICL within the eye, ultimately impacting the vault, which including ICL size and power, anterior chamber depth (ACD), crystalline lens rise (CLR), angle Kappa, iris configuration, surgical technique, postoperative position, intraocular pressure and natural lens movement (28–30). The study showed that ICL diameter and ACD were the most influential factors, a relatively larger ICL diameter and a greater preoperative ACD directly result in higher postoperative vault. In addition, CLR, refers to the anterior movement of the natural crystalline lens within the eye, is also an important factor in ICL surgery (31). CRL can affect the position and movement of the natural lens and the ICL, excessive CLR can lead to potential complications such as contact between the ICL and the natural lens, which may result in elevated IOP. Therefore, accurate preoperative ocular measurements, such as ACD, white-to-white (WTW) distance, angle-to-angle (ATA) distance, angle Kappa, accommodative amplitude, sulcus-to-sulcus (STS) diameter and anterior segment optical coherence tomography (AS-OCT) are very important for preoperative crystal selection and appropriate postoperative ICL vault prediction (22, 32, 33).

**Figure 3:**
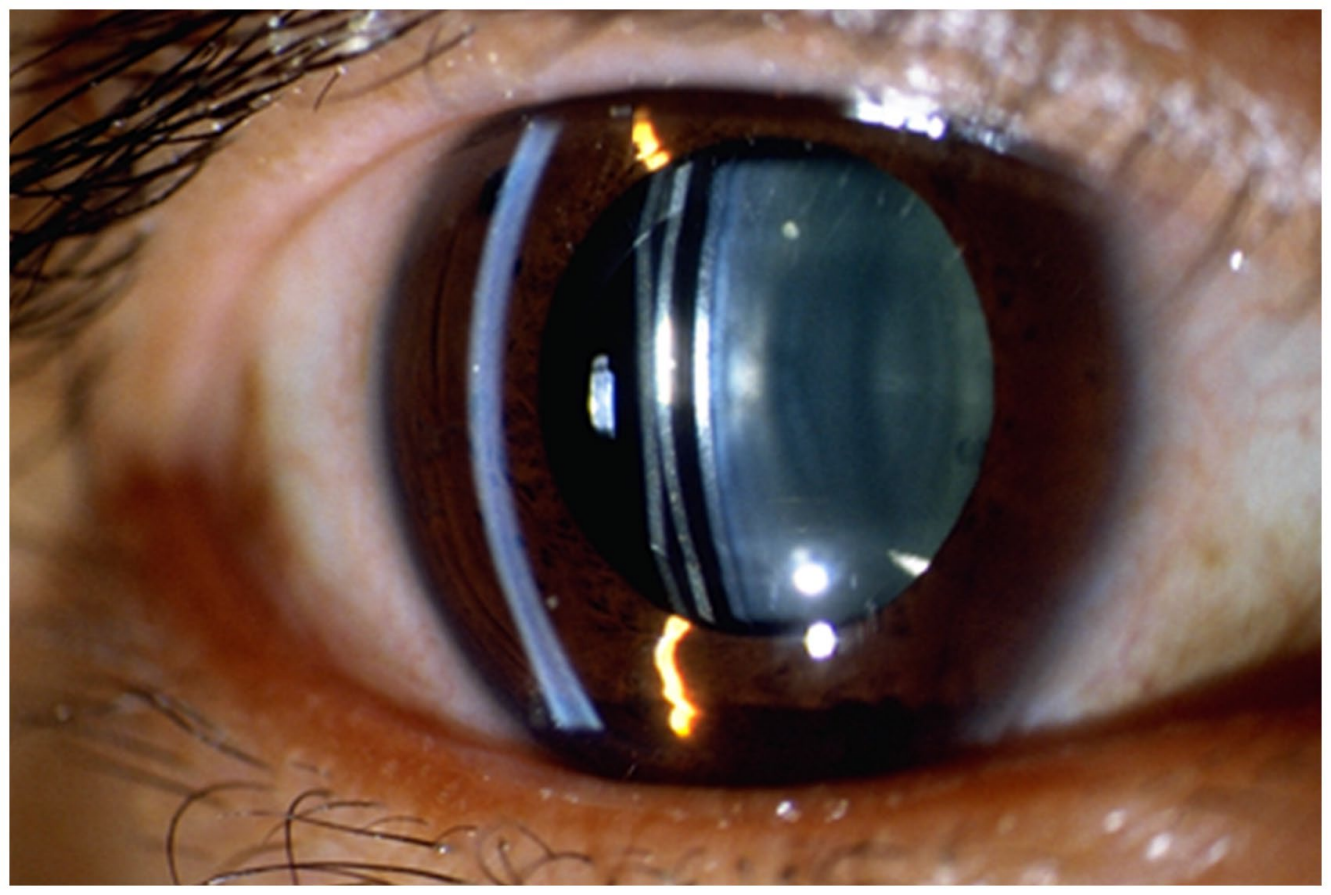
Shows observation of lens vault using slit lamp after ICL implantation.

Management Strategies: Prior to surgery, precise measurements of various anterior segment parameters can be obtained through devices such as AS-OCT, Ultrasound Biomicroscopy (UBM), and Anterior Segment Comprehensive Analyzer (Pentacam). By considering multiple factors and using predictive equations, a reasonable selection of ICL size can be made, greatly reducing the risk of postoperative elevated IOP due to excessive vault. Where device availability permits, intraoperative use of OCT assisted ICL implantation helps to achieve the ideal vault (34). After ICL implantation, regular observation of vault changes using slit-lamp microscopy and AS-OCT can help prevent early elevated IOP and glaucoma, thus avoiding late-stage vision impairment (35). Figure 3 shows observation of lens vault using slit lamp after ICL implantation. Since the axial rotation of the ICL can lead to changes in vault height, the axial variations after ICL implantation should also be considered as indicators for long-term observation. If postoperative vault is excessively high and causes an increase in IOP, timely use of antiglaucoma medications to lower IOP is recommended to prevent further damage to the eyes. Furthermore, if the patient was implanted with a lens that is not a toric ICL, the vault can usually be reduced by rotating the lens in the vertical axis (36, 37). In cases of pupillary block glaucoma, laser iridotomy or iridectomy may be necessary, and if needed, the ICL should be promptly removed.

## 5 Toxic anterior segment syndrome

Toxic Anterior Segment Syndrome (TASS) is a rare and potentially destructive aseptic inflammatory reaction occurring in intraocular surgery. It is associated with the entry of various non-infectious toxic substances into the anterior segment of the eye. Unlike infectious endophthalmitis, this inflammatory reaction is limited to the anterior segment, and Gram stain and bacterial cultures of aqueous and vitreous humor are negative. Known major causes include preservatives in ophthalmic solutions, denatured viscoelastic substances, bacterial endotoxins, and inflammation induced by artificial intraocular lenses (38). Although ICL implantation surgery is less time-consuming and involves fewer steps, the entry of non-infectious toxic substances is difficult to avoid, and there have been occasional reports of TASS occurring after ICL implantation (39–41). TASS typically manifests acutely within 12 to 48 h postoperatively, with some cases exhibiting delayed reactions. Key symptoms include elevated intraocular pressure, corneal edema, and other anterior segment inflammatory reactions. In severe cases, fibrinous exudation in the anterior chamber and even purulent accumulation may occur. The primary mechanism leading to increased intraocular pressure in TASS is the early inflammatory exudate blocking the trabecular meshwork, resulting in elevated intraocular pressure. Some reports also suggest that severe inflammatory reactions triggered by TASS can lead to iris adhesions, causing pupillary block and ultimately resulting in acute elevation of intraocular pressure. TASS may also cause permanent damage to the trabecular meshwork, leading to chronic elevation of intraocular pressure (41).

Management Strategy: The most critical differential diagnosis for TASS is infectious endophthalmitis. The management of TASS should primarily focus on prevention because once toxic substances enter the eye, clinicians have limited measures to address the ensuing inflammatory response. Early diagnosis and treatment of TASS are crucial for maintaining the integrity of eye function and structure, and timely intervention can result in 100% recovery without sequelae. The primary treatment for TASS involves the topical or systemic application of corticosteroids. Corticosteroid eye drops should be instilled every 1–2 h, especially on the first day of onset, to prevent the progression of inflammatory reactions. Routine anterior chamber irrigation should not be performed for severe anterior chamber reactions to avoid exacerbating anterior chamber damage. Daily examinations using slit-lamp microscopy and intraocular pressure measurements are essential. Once intraocular pressure is under control and the cornea becomes transparent, signs of damage to the angle should be examined. The prognosis of TASS depends on the type, quantity, and duration of exposure to toxic substances. If the inflammatory response is mild, recovery can occur within days to weeks. In cases of moderate severity, recovery may take weeks to months, with the possibility of residual corneal edema and mild intraocular pressure elevation. Severe cases may result in permanent corneal opacity and secondary glaucoma, requiring medical or even surgical treatment.

## 6 Urrets-Zavalia syndrome

Urrets-Zavalia Syndrome (UZS) is an unexplained pupillary dilation that occurs after intraocular surgery. UZS is very uncommon, especially in the implantation of the new generation ICL V4c. Patients typically present with symptoms such as glare, halos, and photophobia. On examination, elevated IOP, enlarged pupils, and unresponsiveness to miotic drugs are observed (42). In addition to these manifestations, UZS can lead to angle closure in the anterior chamber, further increasing IOP and causing significant damage to the patient’s eye health (43). Although the syndrome is rare, there have been occasional reports of UZS occurring after ICL implantation (44–46). The exact pathogenesis of UZS after ICL implantation is unclear, but early postoperative elevation of IOP, increased intraocular pressure, and the presence of air or gas in the anterior chamber appear to be significant risk factors for UZS after ophthalmic surgery (42). Immediate control of postoperative elevated intraocular pressure, light responsiveness after dilation, and responsiveness to 2% pilocarpine eye drops have been reported as potential reversible predictive factors for UZS (45). Despite the relatively low reported incidence of UZS, the associated visual symptoms can significantly impact patients’ daily lives, necessitating ophthalmologists to take necessary management measures.

Management Strategy: In cases of short-term pupillary dilation combined with elevated intraocular pressure after ICL surgery, UZS should be considered after common complications have been ruled out. Treatment options for UZS include mannitol, topical application of intraocular pressure-lowering medications, removal of air or gas from the anterior chamber, and, if necessary, iris resection to relieve anterior chamber angle closure and prevent the development of secondary glaucoma (42, 43).

## 7 Pigment dispersion syndrome

Although manufacturers have not reported glaucoma secondary to pigment dispersion as a severe adverse consequence following ICL implantation, occasional cases of PDS after ICL implantation have been documented (47, 48). Pigment Dispersion Syndrome (PDS) is a condition primarily affecting young, myopic adults. The primary mechanism involves the release and deposition of iris pigment in various structures of the anterior segment of the eye. While most patients experiencing pigment dispersion episodes are asymptomatic, extreme photophobia, eye pain, redness, and blurred vision may occur. Other characteristic signs include iris contact, iris concavity, 360° peripheral iris transillumination, increased pigment deposition in the trabecular meshwork, and pigment deposition on the corneal endothelium (Krukenberg spindle). Due to pigment deposition causing trabecular meshwork blockage and reducing outflow facility, there is an increased risk of elevated IOP and pigment dispersion glaucoma (PDG) (49).

The implantation of the ICL can induce pigment deposition in the trabecular meshwork and elevated IOP in the short term, leading to the development of secondary glaucoma in the late postoperative period. This is primarily attributed to laser iridotomy or iris friction with the ICL. However, there are also reports suggesting that the implantation of ICL has no effect on pigment changes in the trabecular meshwork (50). Conversely, the results of a prospective observational study indicate a significant improvement in the morphology of iris concavity in highly myopic patients after EVO ICL implantation, reducing the risk of intraocular pigment dispersion caused by iris concavity (51).

Management Strategy: Given the risk of permanent visual field loss, both patients and healthcare professionals should be aware that PDG is a serious postoperative complication that threatens vision. Due to the dynamic changes in the posterior chamber over time, PDG may occur several years after the implantation of the ICL. Therefore, close monitoring of IOP is essential, and meticulous slit-lamp examinations should be conducted to assess signs of pigment dispersion, including increased pigment deposition observed in the angle during gonioscopy. UBM and AS-OCT can aid in evaluating changes in ICL positioning over time. Once PDG and secondary elevation of IOP occur, prompt management with IOP-lowering and anti-inflammatory treatments is warranted. If necessary, ICL removal should be considered to reduce friction between the lens and the iris. Multiple studies have confirmed the safety and effectiveness of filtration surgery in treating pigmentary glaucoma, but evidence supporting minimally invasive glaucoma surgery for pigmentary glaucoma is currently insufficient (47, 52).

## 8 Malignant glaucoma

Malignant glaucoma is a rare and alarming complication, with few reported cases following ICL implantation (53–55). If not promptly addressed, the condition of malignant glaucoma can persist, eventually leading to corneal decompensation, glaucomatous optic neuropathy, and blindness. The mechanism behind malignant glaucoma is not yet fully understood, and the most widely accepted theory is Shaffer’s proposal that anterior rotation of the ciliary body causes aqueous to accumulate in the vitreous cavity (56). In a study by Senthil et al., researchers suggested that the occurrence of malignant glaucoma after ICL surgery may be due to stimulation and inflammatory reactions to the ciliary body following ICL placement, resulting in anterior rotation of the ciliary body, shallowing of the anterior chamber (AC), and misdirection of aqueous humor into the vitreous cavity (54). There are also reports suggesting that the cause of malignant glaucoma after ICL implantation could be an undersized ICL, leading to congestion of the ciliary body and damage to the zonules, resulting in relatively poor forward flow of aqueous humor, forcing a reverse flow into the vitreous cavity, ultimately forming a malignant cycle (53).

Management Strategy: The diagnosis of malignant glaucoma after ICL surgery is primarily based on clinical manifestations such as postoperative elevated IOP, corneal edema, and anterior chamber disappearance. It is crucial to exclude conditions such as pupil block, excessively high ICL vault, and suprachoroidal hemorrhage. Once malignant glaucoma is confirmed, prompt resolution of aqueous humor blockade is imperative. Drug therapy is the first-line treatment for malignant glaucoma, involving the local use of potent ciliary muscle paralytics, topical anti-inflammatory drugs, and aqueous humor suppressants, coupled with systemic administration of hyperosmotic agents. If conservative treatment proves ineffective, further interventions such as vitrectomy combined with iridotomy or iridectomy, with or without ICL removal, may be necessary (54, 55, 57).

## 9 Discussion

In summary, as a widely used surgical method for myopia correction, ICL implantation still presents various complications, with IOP elevation being a relatively common occurrence. This paper provides a comprehensive analysis of the causes of IOP elevation after ICL implantation and management strategies, as summarized in Table 1. The aim is to offer ICL surgeons an in-depth reference to enhance the safety of the procedure, reduce the risk of complications, and provide patients with better corrective options. Future research could further explore predictive factors for IOP elevation after ICL surgery, safer surgical techniques, and more effective treatment methods to continuously optimize the outcomes of this corrective approach.

**Table 1:** Causes of elevated IOP after ICL implantation and management strategies.

Causes	Clinical manifestation	Management strategies
Residual ophthalmic viscosurgical device	Intolerable ocular pain and corneal epithelial oedema in the first 24 h. Peak intraocular pressure (IOP) is usually 30 mmHg or higher, and residual viscoelastic in the anterior chamber can be observed by slit lamp.	Surgeons can choose the OVD application method based on their own experience. If the IOP is only mildly elevated, short-term application of ocular hypotensive eye drops may be considered. If the IOP exceeds 30 mmHg and continues to rise, it is advisable to consider re-flushing the anterior chamber to thoroughly remove the remaining viscosurgical substance.
Steroid response	IOP elevation usually occurs 1 to 4 weeks after surgery.	Discontinuation or replacement with a lower-potency corticosteroid is recommended, accompanied by the topical application of glaucoma medications. If the intraocular pressure continues to rise, further surgical treatment is required. The most common surgery is trabeculectomy, but implantation of a drainage device or ciliary disruption may also be considered.
Excessive ICL vault	Corneal oedema, anterior chamber shallowness, iridocorneal contact, and imaging findings suggesting an vault>750um can be observed after surgery.	Precise measurement of various anterior segment parameters before surgery, and reasonable selection of ICL model. If conditions permit, OCT can be used during surgery. Use slit lamp microscope and AS-OCT to observe the crystalline arch regularly after surgery. Apply glaucoma medications to reduce IOP in a prompt period of time. If the patient has a lens that is not a toric ICL, the vault can usually be lowered by rotating the lens in the vertical axis. In cases of pupillary block glaucoma, laser iridotomy or iridectomy may be necessary, and if needed, the ICL should be promptly removed.
Toxic anterior segment syndrome (TASS)	Acute manifestation within 12–48 h after surgery, with the main symptoms including elevated IOP, corneal oedema and other anterior segment inflammatory reactions	Excluding infectious endophthalmitis The main method is topical or systemic application of steroids. The routine anterior chamber flushing should not be carried out, which may aggravate the damage of the anterior chamber.
Urrets-Zavalia syndrome (UZS)	Early postoperative symptoms such as glare, halos and photophobia, signs such as increased intraocular pressure, pupil dilation and unresponsiveness to pupillary medication can be observed.	Mannitol, topical use of drugs to reduce intraocular pressure. Remove air or gas from the anterior chamber. If necessary, iridectomy can be conducted to alleviate the closure of the anterior chamber angle.
Pigment dispersion syndrome (PDS)	Induced in the short-term postoperative period, may present with extreme photophobia, ocular pain, eye redness and blurred vision, with signs including iris contact, iris concavity, 360° peripheral iris transillumination, increased pigment deposition in the trabecular meshwork, and pigment deposition on the corneal endothelium (Krukenberg spindle).	Detailed slit lamp examination to assess for signs of pigment dispersio. IOP lowering and anti-inflammatory therapy should be performed as soon as PDG and secondary IOP elevation are present. Consider removal of the ICL if necessary to reduce friction between the lens and the iris. Filtration surgery is safe and effective in the treatment of pigmentary glaucoma.
Malignant glaucoma	Elevated IOP, corneal edema and anterior chamber disappearance occurred in the early postoperative period.	exclude conditions such as pupil block, excessively high ICL vault, and suprachoroidal hemorrhag. Pharmacological treatment is the first line of treatment for malignant glaucoma, including topical potent ciliary muscle paralysers, topical anti-inflammatory agents and aqueous humor inhibitors, as well as systemic hypertonic agents. If conservative treatment is not effective, further surgical intervention is required, such as vitrectomy combined with iridotomy or iridectomy, with or without removal of the ICL.

## Author contributions

DG: Writing – original draft, Writing – review & editing. SD: Conceptualization, Investigation, Writing – original draft. KD: Conceptualization, Writing – original draft. ZY: Project administration, Supervision, Writing – review & editing. JW: Funding acquisition, Project administration, Supervision, Writing – review & editing.
